# Smoothness without Smoothing: Why Gaussian Naive Bayes Is Not Naive for Multi-Subject Searchlight Studies

**DOI:** 10.1371/journal.pone.0069566

**Published:** 2013-07-26

**Authors:** Rajeev D. S. Raizada, Yune-Sang Lee

**Affiliations:** 1 Dept. of Psychology, Cornell University, Ithaca, New York, United States of America; 2 Psychological and Brain Sciences, Dartmouth College, Hanover, New Hampshire, United States of America; 3 Center for Cognitive Neuroscience, University of Pennsylvania, Philadelphia, Pennsylvania, United States of America; Beijing Normal University, China

## Abstract

Spatial smoothness is helpful when averaging fMRI signals across multiple subjects, as it allows different subjects' corresponding brain areas to be pooled together even if they are slightly misaligned. However, smoothing is usually not applied when performing multivoxel pattern-based analyses (MVPA), as it runs the risk of blurring away the information that fine-grained spatial patterns contain. It would therefore be desirable, if possible, to carry out pattern-based analyses which take unsmoothed data as their input but which produce smooth images as output. We show here that the Gaussian Naive Bayes (GNB) classifier does precisely this, when it is used in “searchlight” pattern-based analyses. We explain why this occurs, and illustrate the effect in real fMRI data. Moreover, we show that analyses using GNBs produce results at the multi-subject level which are statistically robust, neurally plausible, and which replicate across two independent data sets. By contrast, SVM classifiers applied to the same data do not generate a replication, even if the SVM-derived searchlight maps have smoothing applied to them. An additional advantage of GNB classifiers for searchlight analyses is that they are orders of magnitude faster to compute than more complex alternatives such as SVMs. Collectively, these results suggest that Gaussian Naive Bayes classifiers may be a highly non-naive choice for multi-subject pattern-based fMRI studies.

## Introduction

When using pattern recognition algorithms to analyze fMRI data, it might be expected that the classifiers which are most powerful at the single-subject level will also yield the best results at the multi-subject group-level. However, at the group level, a new question arises which does not apply when analyzing a single individual, namely, that of how best to combine information across multiple subjects. In the present paper, we show that the GNB classifier has properties which make it particularly well-suited for analyzing multi-subject studies. We do this by using theoretical arguments and also evidence from two independent fMRI data sets.

The machine learning literature contains many comparisons of classifier performance, across many domains [1]. However, when using classifiers to analyze fMRI data, problems arise which are specific to neuroimaging, and which have therefore not been addressed in those domain-general studies. In particular, multi-subject fMRI studies must deal with imperfections in the process of normalizing subjects' brains to a common space. A given brain structure may occupy a specific voxel coordinate in one subject, but it may lie in a nearby but different voxel in a different subject. Voxel-wise averaging across subjects will therefore fail to average those corresponding brain signals together with each other, because their coordinates, although close together, are not identical.

In standard GLM analyses, subjects' brain images have spatial smoothing applied to them before inter-subject averaging. This smoothing helps with inter-subject alignment because the signal from a given voxel in one subject will now be averaged together not only with the signal from the exactly corresponding voxel position in the other subjects, but also with the signal from the voxel's nearby neighbors. Smoothed images are likely to give rise to more sensitive group-level inference, as has been shown by Refs [2], [3]. However, it should be also noted that spatial smoothing does not always improve group-level inference: for example, as discussed by Li and colleagues [4], there can be circumstances in which smoothing is helpful only when it is applied at adaptively varying spatial scales in order to take into account the shape and spatial extent of specific regions of interest.

In multivoxel pattern-based analyses, the fMRI data is typically not smoothed, as such smoothing would run the risk of blurring away the information that fine-grained spatial patterns contain. By taking unsmoothed data as their input, pattern-based analyses, unlike GLM analyses, do not inherit the benefits of smoothness for dealing with spatial misalignment across subjects.

This problem of spatial misalignment might not at first seem relevant to the choice of classifier, as the process of normalizing brain data to a common space is distinct from that of applying a classifier to the normalized data. However, if subjects' brain images are spatially smooth, then corresponding brain activations will be combined across subjects even when they are slightly misaligned. (It is worth bearing in mind, however, that subjects' brains can sometimes be so misaligned that smoothing will fail to help, e.g. when their brains have lesions or severe atrophy). This helpful role of smoothness is where the choice of classifier comes in, for the following reason: the output images of pattern-based analyses which use GNBs turn out to be smooth. Below, we present empirical evidence for the claim that GNBs produce smooth images, and give a theoretical explanation of why this occurs. The type of analysis in which GNBs lead to smoothness is a widely used and increasingly popular method of conducting multivoxel pattern-based fMRI studies: searchlight analysis [5].

### 1. Searchlight analyses for information-based fMRI

In standard fMRI analyses, the numbers inside voxels that are averaged together across subjects are activation values: the degree to which each voxel increases or decreases is signal intensity evoked by a given task or stimulus condition. By contrast, searchlight analyses write a number into each voxel which is a measure of classification in that voxel's local neighborhood. After searchlight analyses are complete for individual subjects, these output maps are then entered into second-level random-effects analyses, in the same way as is done with single-subject beta-coefficient images from standard General Linear Model (GLM) analyses.

### 2. Overlapping searchlights, covariance, and smoothness

The fMRI data that get entered into searchlight analyses are usually unsmoothed, as this allows the classifier applied to the voxels within each searchlight neighborhood to seek information in the fine-scale “salt and pepper” spatial patterns that smoothing would have acted to remove (But see also Ref [6]).

However, the fact that unsmoothed data go in does not necessary mean that non-smooth information maps come out. One factor which tends to create some smoothness in searchlight maps, regardless of which type of classifier is used, is the high degree of spatial overlap between the searchlight neighborhoods of contiguous voxels. For example, consider a neighborhood whose radius is three voxels in size, centered on a given voxel. The information value that will get written as analysis-output into that voxel is a function of the voxels in that particular neighborhood. As we move to a voxel immediately abutting this one, and now use the contents of this new voxel's neighborhood as input for the classifier, many of the voxels in the new voxel's neighborhood will also have been in the old one. In moving from one searchlight neighborhood to the next, a one-voxel wide shell of voxels will have been shaved off from the trailing edge of the old searchlight, and similarly a one-voxel wide shell on the opposite side will have been added. But the central portions of the two voxels' neighborhoods will completely overlap.

Because the output of a classifier is a function of which voxels' data are entered into it, the information extracted from one searchlight neighborhood and the information extracted from its overlapping neighbor will tend to be similar. In other words, the output value in one voxel will tend to be close in value to that of its neighbor, and that is simply to say that the information map will tend to be smooth.

However, the degree of smoothness can vary greatly, depending upon what kind of classifier is used. Below, we demonstrate this using real data, but before that we also present a heuristic explanation. A key factor is whether or not the classifier takes the covariance between the voxels' signals into account. The most commonly used type of classifier which does *not* take covariance into account is Gaussian Naive Bayes (GNB). In the following sections, we describe how a GNB classifier works, how it can perform surprisingly well despite the fact that it does not model covariance, and then how the very fact that it does not model covariance causes GNB-produced searchlight maps to be smooth. Finally, we validate our proposed use of GNB classifiers by showing that they lead to replicated and interpretable results across two independent data sets.

### 3. Gaussian Naive Bayes: what it is, and some strengths and weaknesses

**Figure 1:**
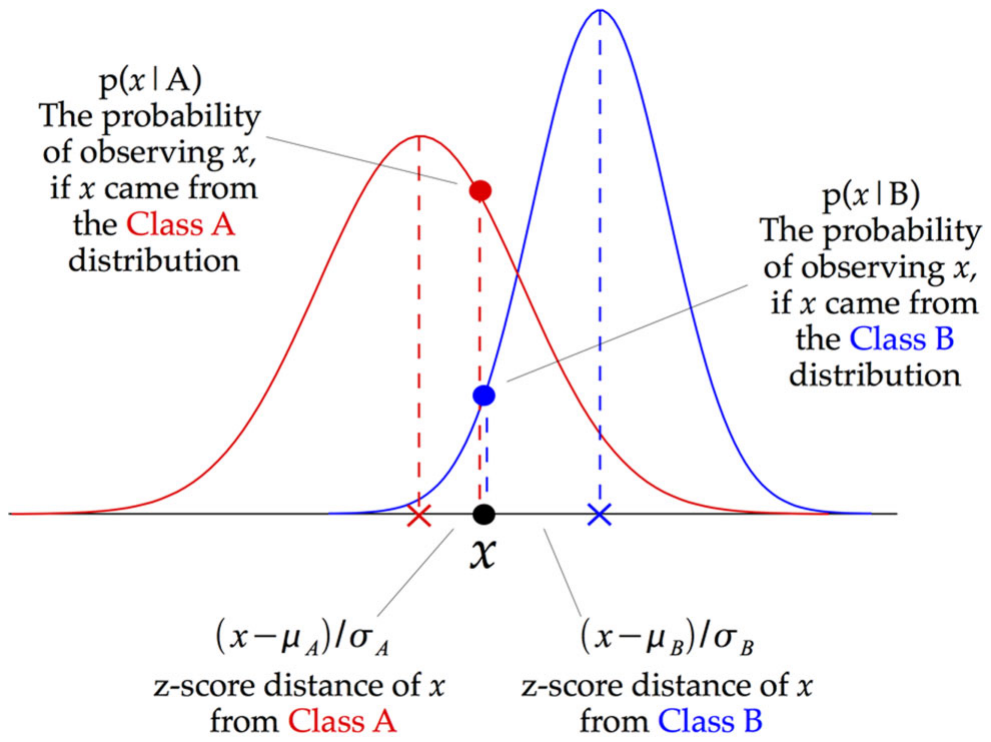
Illustration of how a Gaussian Naive Bayes (GNB) classifier works. For each data point, the z-score distance between that point and each class-mean is calculated, namely the distance from the class mean divided by the standard deviation of that class. Note that this schematic just shows one dimension, whereas a crucial distinction between GNBs and other classifiers arises only when there is more than one input dimension: the GNB does not model the covariance between dimensions, but other types of classifier do.

Figure 1 illustrates how a Gaussian Naive Bayes (GNB) classifier works. In essence, the approach takes each data point, and assigns it to whichever class it is nearest to. However, rather than calculating that nearness by using Euclidean distance from the class-means, the GNB takes into account not only the distance from the mean but also how this compares to the class variance. For each dimension (in the figure, just one dimension is shown), the z-score is calculated, namely, the distance from the mean divided by the standard deviation. In Fig. 1, the z-score distance from Class A is written as 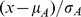, with the z-score distance from Class B being labeled similarly.

The “Gaussian” part of Gaussian Naive Bayes now comes in. The classifier makes the assumption that the classes have Gaussian normal distributions. This allows each z-score distance to be converted directly into a p-value, as illustrated in Fig. 1. This p-value is the probability of observing a given data point, *x*, if *x* were drawn from the distribution of a particular class. However, what we want is not the probability of the data given a particular class, but instead the probability of a class, given our observed data. That is where the “Bayes” part of Gaussian Naive Bayes now comes in, as Bayes' Theorem allows us to derive each one from the other.

The weaknesses, but also some surprising strengths, come from the “Naive” part of Gaussian Naive Bayes. The naive aspect of the algorithm is that it treats all of the input dimensions as independent from each other. Another way of saying this is as follows: even if there is covariance between two or more input dimensions, the GNB classifier does not model it.

**Figure 2:**
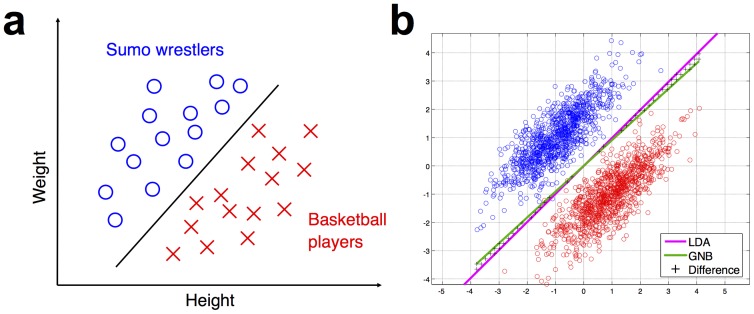
Basic illustration of how a GNB classifier can perform well in a categorization task, even when there is task-relevant covariance between the input dimensions. The task, showing in panel (**a**) is to distinguish between sumo wrestlers and basketball players, based on the input dimensions of height and weight. Only considering one dimension at a time is insufficient to perform the categorization. However, as panel (**b**) illustrates, the classification boundary drawn by a GNB (shown in green) is almost identical to that drawn by linear discriminant analysis (LDA, shown in purple). The two different classifiers give different class predictions only in a very small part of the input space, marked with black crosses. The LDA classifier is Fisher's Linear Discriminant, which is similar to a GNB in that it models the mean and variance of the data's input dimensions, but different in that it also models the covariance of the dimensions.

To see why this might cause problems (although we will see shortly that those problems are often much less severe than might at first be expected), consider the cartoon example shown in Figure 2a, which illustrates the point by using the stimulus dimensions of height and weight to distinguish between sumo wrestlers and basketball players. It is clear that these input dimensions allow the classification task to be performed successfully. However, note that no individual dimension on its own is sufficient to separate one category from the other. It is necessary to take both height and weight into account, as evidenced by the fact that the dividing class boundary is diagonal, rather than vertical or horizontal. In other words: the heights and weights of these data points are not independent, but instead have a positive covariance. The weakness of a GNB classifier is that it does not consider this joint distribution of height and weight. It can only model each dimension separately.

### 4. Gaussian Naive Bayes classifier vs. Gaussian smoothing

It may be helpful at this point to highlight a crucial distinction which might otherwise cause confusion. The classifier that we are discussing in this paper uses Gaussians, hence its name Gaussian Naive Bayes, and we are arguing that when used in conjunction with the searchlight technique it produces smooth output images. Gaussians are also often used in standard fMRI analyses in a different sense, also related to smoothing: BOLD images are typically preprocessed by being spatially smoothed with a Gaussian, typically about 8 mm in width. These two uses of Gaussians are completely different, as we explain immediately below, but, given the overlap in terminology, it would be easy and quite understandable to confuse between them.

The Gaussian in the GNB classifier is a probability distribution, and has the effect of comparing neural activation to the means and variances of activation in different stimulus conditions. The output of the classifier is a condition-label. By contrast, the Gaussian in spatial smoothing is not related to probabilities, but instead is a physical region across which voxels values are weighed, summed and averaged. The output of spatial smoothing is not a condition label, but is instead an average activation value.

A potential source of confusion here is the following: although the GNB classifier yields condition-labels as outputs, the searchlight technique does produce 3D volumes as outputs. Each voxel in a searchlight output-image is not an activation value, but instead is the accuracy value achieved by entering the activation patterns in the local spatial neighborhood contained in the searchlight sphere into a classifier. Such output images often tend to be smooth, due to the fact that neighboring searchlight spheres contain overlapping sets of voxels.

Critically, however, saying that a searchlight output volume is smooth is not at all the same thing as saying that the output volume is derived by spatially smoothing the input activation patterns. The voxel-values in searchlight output volume are determined by the input activation patterns. These input patterns could have very large activation values but the output value in the searchlight volume could be very low, if the activation patterns for different stimulus conditions are not separable. Conversely, very low-intensity input activation values can yield high searchlight volume voxel-values, if the input activation patterns are separable and the classifier is therefore highly accurate at discriminating between those patterns. In summary, despite the potentially confusing facts that both operations involve the word “Gaussian” and that both approaches produce smooth output images, the use of a Gaussian Naive Bayes classifier in a searchlight analysis and the application of Gaussian spatial smoothing to an image are completely different procedures.

### 5. Why naive Bayes may not be so naive a classifier after all

Given the covariance between height and weight in our sumo wrestler vs. basketball player example above, it might be expected that a GNB classifier would perform very poorly on this dataset. However, as Figure 2b shows, the classification boundary drawn by a GNB (shown in green) is almost identical to that drawn by linear discriminant analysis (LDA, shown in purple). The two different classifiers give different class predictions only in a very small part of the input space, marked with black crosses. The LDA classifier is Fisher's Linear Discriminant, which is similar to a GNB in that it models the mean and variance of the data's input dimensions, but has the key difference that it also models the covariance of the dimensions.

That covariance is precisely what the GNB does not model. How, then, does the covariance-ignoring GNB end up drawing almost exactly the same decision boundary as the covariance-modeling LDA? Several articles in the machine learning literature have highlighted the fact that GNB classifiers often perform surprisingly well, and have explored a variety of statistical factors that underlie this. For the detailed technical arguments, the reader is invited to refer to that literature [7]–[11]. Here we present a summary of one of the key points, illustrating the argument with diagrams inspired by Ref [11].

The distances which a GNB classifier calculates, illustrated in Fig. 1, are distances from class-centers. Whichever class-center a particular data point is closest to will be the class to which that point will be assigned. The GNB is not calculating plain Euclidean distances to the class-centers, but instead normalizes the distance along each dimension by the variance along that same dimension.

Assigning each data point to the class whose center is nearest will produce a decision boundary which lies halfway between the two class centers, and which is perpendicular to the line joining those centers (this line is referred to in geometry as the perpendicular bisector). This decision boundary will make for a successful classifier, unless the classes themselves are shaped such that they cross over it.

**Figure 3:**
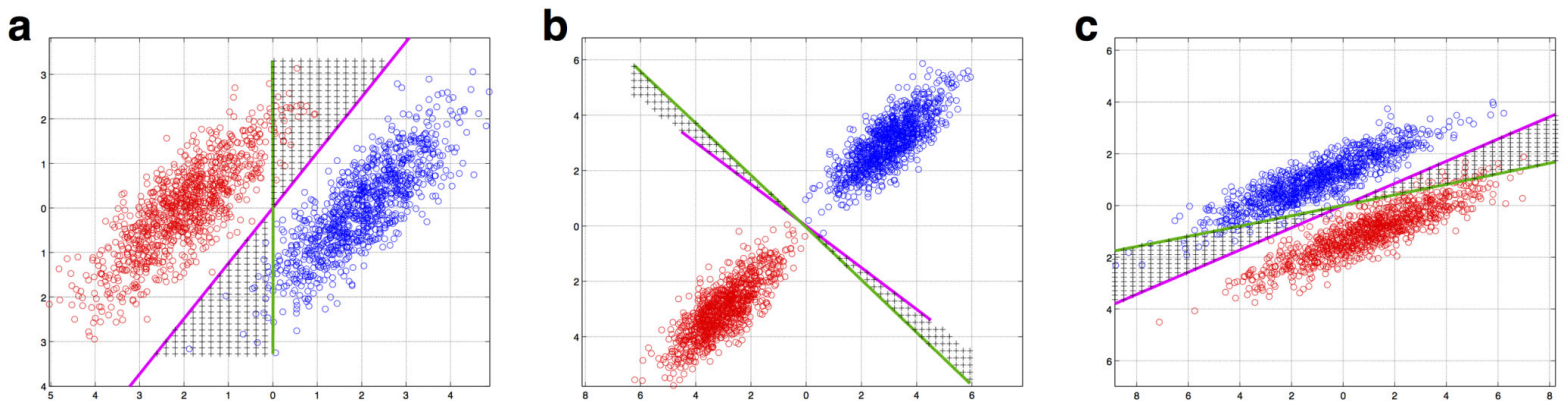
Some examples where covariance does actually hurt the GNB's performance. These were made by shifting the class centers, such that their perpendicular bisector is no longer parallel to the direction of maximal covariance. The regions of black crosses show where the covariance-ignoring GNB (green line) and the covariance-modeling LDA (purple line) yield different predictions. Nonetheless, these regions still form a relatively small proportion of the overall input space.

As can be seen from Fig. 2b, the sumo and basketball classes *do not* tend to cross over the GNB's decision boundary. The covariance between the input dimensions of weight and height has the effect of stretching out the shape of the classes into long and thin ellipses, but those ellipses are stretched out in the same direction as the boundary defined by the class centers. (In geometrical terms: the direction of maximal covariance runs almost parallel to the perpendicular bisector of the class centers). Of course, we can also consider situations where the covariance does hamper the GNB's performance. Figure 3 illustrates some examples where the class centers are shifted such that their perpendicular bisectors are no longer parallel to the direction of maximal covariance. Nonetheless, even in Figure 3a, this region forms a relatively small proportion of the overall input space. Together, the GNB classifier may not be so naive after all in that prediction is not hugely affected by disregarding covariance structure.

### 6. Modeling covariance does not always help as much as might be expected: getting a good estimate is hard

The arguments above highlight the perhaps surprising fact that the GNB can perform well, despite the fact that it does not model the covariance between data dimensions. A converse argument makes a different but supporting point: although non-GNB classifiers (e.g., LDA) can seek extra predictive power by modeling covariance, they will be able to improve their performance only insofar as they succeed in modeling the covariance accurately. However, it is often quite difficult to get an accurate estimate of the covariance of a data set, especially when the data are high-dimensional. In a *p*-dimensional dataset, the covariance matrix is *p*-by-*p* in size. A GNB classifier only needs to estimate the variances, which are the *p* elements of the matrix's leading diagonal. In order to estimate the full covariance matrix, we need enough data to specify all *p* ^2^ elements of the matrix. The problem is actually more difficult even than that, as classifier algorithms typically require the inverse of the covariance matrix. When the data are very high-dimensional, as is often the case in fMRI data with its thousands of voxels, there may be fewer data points than there are dimensions. When this is the case, the covariance matrix cannot be inverted, and some kind of regularization procedure must be used.

Although regularization allows an estimate of covariance to be made, it does not guarantee that the estimate will be a good one. The resulting estimate is no longer purely a function of the dataset itself, but instead is a mixture of the data and the extra ingredient of the regularization term. This difficulty, which arises very frequently in statistical pattern recognition, is known as the “small sample size” problem [12]. A good overview of methods attempting to tackle it can be found in the final chapter of Ref [13].

### 7. Why smooth output arises from not modeling covariance

As was described above, the searchlight neighborhoods of contiguous voxels are highly overlapping. Given this, it might be expected that the classification scores entered into contiguous voxels would therefore be similar. In other words, it might be expected that searchlight information maps would be smooth.

However, the covariance between the fMRI signals in a set of voxels can change quite markedly when the membership of that set is changed, even if the majority of the voxels maintain their membership in the set.

The following heuristic example may help to illustrate this. Consider that the members of a research lab are meeting in a room, and our task is to count up the total number of emails sent in the preceding week by all of the room's occupants. If one person now leaves the room and a new person enters to replace them, the total measure of numbers of emails sent will typically not be altered by much.

This tallying of the total numbers of sent emails is like modeling the individual activations of a set of voxels, while not modeling the covariance between them. Now instead, consider the case where we do model the covariance. Instead of just counting how many emails each person has sent, we now make a count of how many emails each person has sent to every other person in the room. This count of pairwise email interactions between the room's occupants is, in effect, the covariance matrix of that lab's internal email correspondence.

If a new person now walks into the room, this email covariance count might change by a small amount or it might change by a large amount, depending on who the new person is. If the new person is a visitor from outside the lab who has exchanged email with only one or two of the lab-members, then the count of pairwise email interactions will stay largely unchanged. However, if the new person is the head of the lab, then they are likely to have exchanged several emails with many of the room's occupants. The addition of this new person to the set makes a large and abrupt change to the set's overall covariance structure.

Returning from the above metaphor to actual fMRI voxels, the above argument suggests that as we move from a searchlight neighborhood centered at one voxel to a searchlight centered at a contiguous voxel, the output of a classifier which does not model covariance would be likely to change more smoothly than would the output of a classifier which does model covariance.

### 8. Tests of the approach's validity, using empirical data

Above, we laid out theoretical arguments for why the GNB classifier may be well-suited for conducting multi-subject searchlight analysis studies. In the Results section below, we present empirical analyses, verifying the theoretical arguments using real fMRI data.

A range of different empirical tests can be used, from the basic to the more complex. At the most basic level, we can simply check whether or not it is the case that the GNB classifiers actually do produce smooth searchlight maps. If that is the case, the group-level inference is likely to improve, given that smooth images yield more sensitive group-level inference [2], [3].

However, we can move beyond an indirect argument about the types of images that ought to aid group-level inference: we can simply carry out the group-level analyses and then assess whether the GNB classifier produces good results. This immediately raises the question of what we should count as “good results” when we are analyzing real empirical data, as there is no predetermined “ideal activation map” which the results must match. The results should, at least, be able to allay two possible concerns: first, there should be robust group-level activation. This would allay the concern that GNB classifiers might be too weak to detect any neural signals. Second, and more qualitatively, the group-level information-bearing activation should be cognitively and neurally plausible. To give an obvious example, finding speech-related activation in Broca's area (as we do in the present data) would be a result with high neural plausibility. Finding speech-related activation in the amygdala would be less so. If our GNB analyses produce plausible results, this would suggest that the patterns are functionally meaningful rather than just being noise.

Moving beyond that, the strongest and most objective test of the approach's validity is whether it can produce replicated results across independent data sets. In the present paper, we show that GNBs does precisely do this, but that SVMs do not.

## Methods

### 1. Two independent data sets, using different task designs but the same stimuli

The analyses were conducted using data from two independently collected data sets: those from Raizada & Poldrack (2007) [14] and Lee et al. (2012)[15]. These two data sets were collected some years apart, with different subjects at different institutes. Critically, these data differed in task designs; for example, the Raizada & Poldrack (2007) experiment used an event-related design that was optimized for conducting an adaptation-fMRI study, whereas the Lee et al. (2012) experiment used a simple block design which was more directly amenable to pattern-based fMRI analysis. The Raizada & Poldrack (2007) study presented stimuli in pairs of two types: identical pairs, i.e. one particular stimulus on the 10-step /ba/-/da/ continuum presented twice in succession (e.g. 4-then-4), and 3-step pairs, in which the two stimuli were three steps apart along the continuum, e.g. 4-then-7. The two stimuli within each pair were separated from each other by 500 ms of silence. In Raizada & Poldrack (2007), the comparison of interest was between the 3-step pairs and the identical-pairs. In Lee et al. (2012), only the identical-pairs were considered.

The details for that Raizada & Poldrack (2007) study were as follows. There were 12 subjects (7 females; age range 21–36). A Siemens 3T Trio scanner at the MGH-NMR Center was used, with a standard EPI BOLD pulse sequence and a clustered volume acquisition with the following parameters: TR = 4 s, TA = 1.8 s, silent gap = 2.2 s, 500 ms interval between stimuli and scanner-noise onset/offset, 25 slices, 3.1×3.1 mm within-plane resolution, 5 mm thick slices with a 0.5 mm skip, and descending slice-ordering. Each stimulus pair was presented in the middle of the 2.2 s clustered volume acquisition silent gap. In the scanner, sounds were played via non-magnetic Koss electrostatic headphones, adapted for fMRI by Giorgio Bonmassar and Patrick Purdon. The fMRI scans were subdivided into 7 runs, with 104 volume acquisitions per run. There were 480 phoneme trials, 20 per type (24 types, 10 same-phoneme, 14 phoneme pairs 3-steps apart), and 100 null trials consisting of silence.

The Lee et al. (2012) details were as follows: there were 13 subjects (9 females; age range 19–34 years). A Philips Achieva 3T whole body scanner was used at Dartmouth College, with a standard EPI BOLD pulse sequence and a clustered volume acquisition with the following parameters: TR = 3 s, TA = 1.88 s, silent gap = 1.12 s, 560 ms interval between stimuli and scanner-noise onset/offset, 32 slices, 3×3 mm within plane resolution, 4 mm thick slices with a 0.5 mm skip, and interleaved slice-ordering. Each stimulus (single stimuli 300 ms long, as opposed to the pairs of stimuli in the 2007 study) was presented in the middle of the 1.12 s clustered volume acquisition silent gap. In the scanner, sounds were played via high-fidelity MR compatible headphones (MR Confon, Germany). The fMRI scans were subdivided into 5 runs, with 185 volume acquisitions per run. A block design was used, with one of the 10 phonemes repeatedly presented five times during the silence gaps during each block. Between the blocks were rest periods lasting 15 s (5 TRs). The ordering of the stimulus blocks was pseudo-randomly generated and was counter-balanced across subjects. There were 18 stimulus blocks per run, making 90 blocks in all across the five runs. Of these, 10 contained quieter catch-trials, and the data from them was not used in subsequent analysis. The remaining 80 blocks consisted of 8 blocks for each of the 10 stimuli along the /ba/-/da/ continuum.

### 2. Commonalities across both studies: stimuli, clustered volume acquisition, alertness task, and psychophysical testing

Both studies used a set of ten stimuli spread along the /ba/-/da/ continuum. Each sound lasted for 300 ms. The stimuli were made using a SenSyn Klatt Synthesizer (Sensimetrics, Inc.) and varied in the 2nd and 3rd formants. Full details of the formant transitions and other synthesis parameters are provided in Ref [14].

During the fMRI scans, both studies used clustered volume acquisition protocols, such that there was a brief silent gap at the end of each brain-volume's-worth of slice acquisitions. This silent gap was long enough for the phoneme stimuli to be played in the middle with brief silent pauses immediately before and after, thereby preventing auditory masking. In both studies, the subjects performed a non-phonetic alertness task during the scans: the task was to listen for occasional quieter catch-stimuli and to press a button when such quieter trials were heard. The fMRI data from these quieter and button-press trials were not used in the subsequent analyses.

After the MRI scan, subjects were psychophysically tested outside of the scanner, in order to determine each subject's perceptual category boundary. The subjects were presented in turn with multiple instances of all ten of the stimuli, randomly interleaved, and they had to identify each one as either /ba/ or /da/. This allowed us to find each individual subject's perceptual category boundary. As above, for full details of the psychophysical testing, see Ref [14]. All subjects, for both studies, were right-handed native English speakers. Both studies were approved by the Committee for the Protection of Human Subjects at Dartmouth College and Massachusetts General Hospital.

### 3. Pattern-based fMRI analysis methods

The MRI scans for Lee et al (2012) [15] were submitted into the pipeline of motion-correction and spatial normalization using SPM8 [16]. While we used unsmoothed data for Lee et al (2012), we had to use smoothed data (6 mm FWHM) for Raizada & Poldrack (2007) due to unfortunate loss of the original raw MRI scans. This was a concern to us, as this smoothing could possibly have erased the spatial pattern information that we were hoping to extract in our pattern-based analyses. However, we were pleasantly surprised that this spatially smoothed data not only provided good pattern-based analysis results, but that these results replicated so closely the analysis of Lee et al. (2012), which was unsmoothed. The apparently harmless nature of the smoothing in Raizada & Poldrack (2007) may be due to the fact that the 6 mm Gaussian kernel was relatively small, compared to original acquisition resolution of that data at 3.1×3.1×5.5 mm. Nonetheless, our finding perhaps adds support to the suggestion made by Op de Beeck in Ref [6] that spatial smoothing may be less detrimental to pattern-based analysis than had been originally believed.

The voxels' time-courses were extracted, and were high-pass filtered with a 300 s cut-off, in order to remove slow drifts. No low-pass temporal whitening filter was applied. Each voxel's time-course was then zero-meaned. For each voxel in the brain, the local spatial neighborhood of voxels was extracted, using a discrete sphere with a radius equal to three-voxels, creating searchlights or “spheres of information” with up to 123 voxels. For centers whose searchlights fell partly outside the SPM-created brain-mask, only the within-mask voxels were used.

Using each subject's /ba/-/da/ phonetic boundary, as psychophysically measured outside of the scanner, the time-points corresponding to /ba/ and to /da/ were calculated by convolving the base condition time-course by an HRF, and then picking those time-points where the convolved result exceeded its mean value. The activation vectors corresponding to the spatial patterns within a given searchlight during each conditions' time-points were then passed into a classifier: either a Gaussian Naive Bayes classifier (GNB), Fisher Linear Discriminant Analysis (LDA), or a linear Support Vector Machine (SVM). The GNB classifier was custom-coded in Matlab, and for the SVM we used the Matlab implementation of a Lagrangian SVM provided by Mangasarian & Musicant [17]. Leave-one-run-out cross-validation was used for all analyses. After individual searchlight maps were acquired, the group-level inference was made as follows: First, for an individual searchlight map, the chance-level score (50%, for the binary classification) was subtracted from a particular prediction score (e.g., 58%) stored in every voxel, which was then averaged across voxels. Then, the average score was subtracted from each voxel's score, resulting in baseline-correction (i.e., the average score was converted to 0) across subjects. Finally, this adjusted-score map was submitted to a random effects analysis. A voxel-wise threshold of *p<0.001* (uncorrected) was used, and the resulting cluster size was corrected for multiple comparisons using both FWE (Family-Wise Error) and FDR (False Discovery Rate).

### 4. Gaussian Naive Bayes classifier implementation

The essentials of the GNB algorithm are illustrated in Fig. 1. We implemented it in Matlab. The equations governing the algorithm are as follows:

Let us call the two conditions to be discriminated between Condition A and Condition B. For each voxel considered individually, the z-score distances of a given data point from the center of Condition A are calculated as follows: 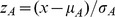, where 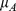 is the mean activation during the time points belonging to Condition A, and 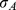 is the standard deviation. The z-score distances from the center of Condition B are calculated similarly. Each z-score is then transformed into a probability value, according to the equation for a Gaussian normal distribution. This p-value is the probability of observing data point *x*, if *x* were a member of the class, in this case Class A:
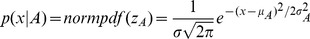
The above equation is calculated separately for each voxel within the searchlight neighborhood. Because the GNB classifier assumes statistical independence between the voxels, the joint probability across all of the voxels is simply the product of the individual probabilities in each voxel. However, multiplying a large number of small p-values together leads to computer rounding-errors. For that reason, and because adding log-probabilities is equivalent to multiplying the actual probabilities, the log p-values are computed and these log-probabilities are added together instead. If there are *n* voxels in the searchlight neighborhood, indexed by *i*, the resulting equations are:
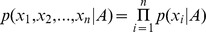


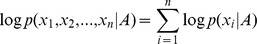
In our analyses, we made sure that equal numbers of data points were entered into the training set from Class A and Class B, by excluding a subset of data points from one of the classes if the numbers were imbalanced. This allowed us to use equal prior probabilities for the two classes. Given this, each data point in the testing set could be assigned to either Class A or Class B simply by determining which of 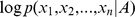 and 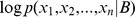 was greater.

### 5. Calculation of Fourier power as a measure of smoothness

**Figure 4:**
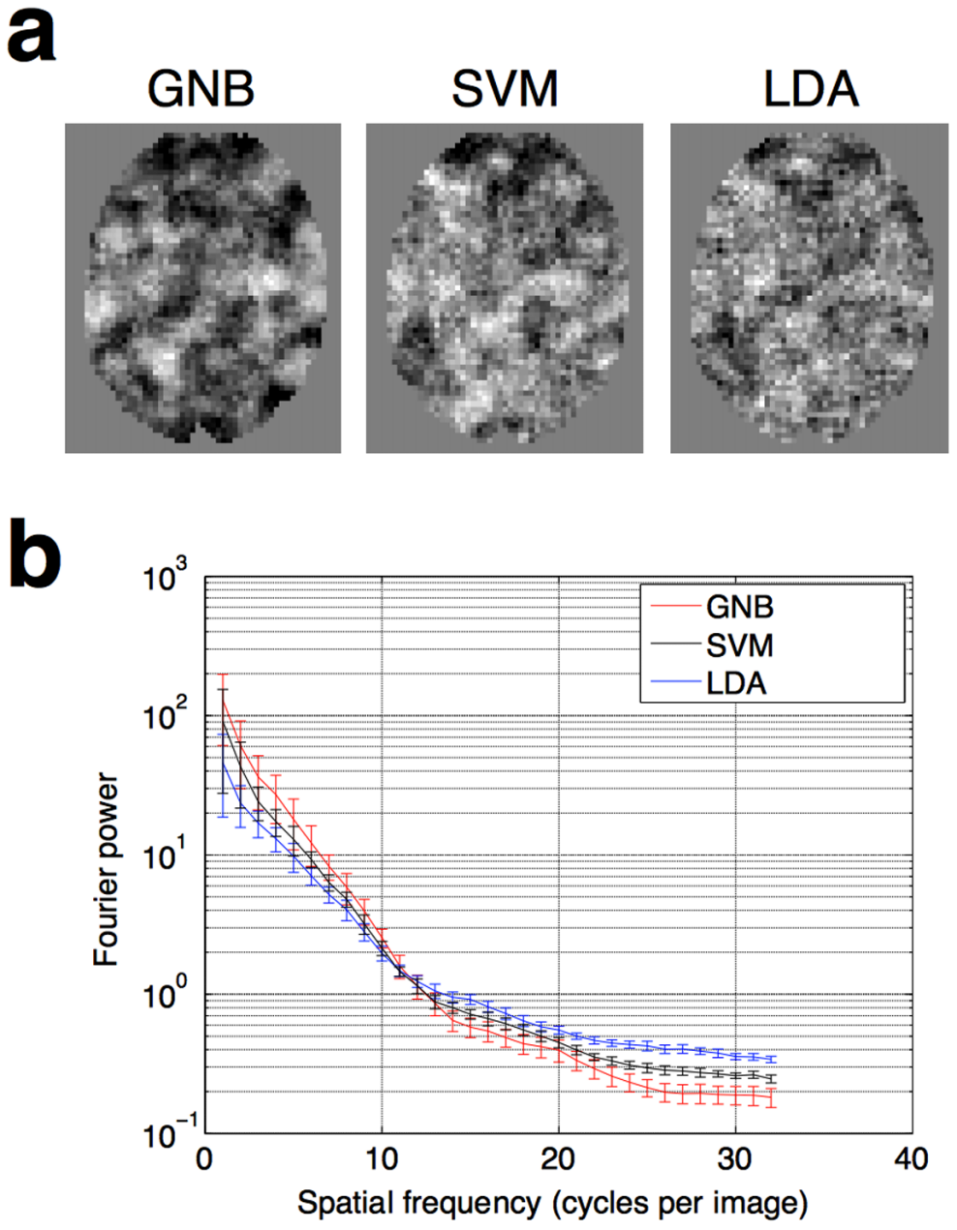
Comparison of the smoothness of searchlight maps generated by different classifiers. (**a**) Illustrative slices drawn from one individual. It can be seen from a simple visual comparison that the smoothest information maps arise from using GNB classifiers, which do not model covariance. (**b**) **A quantitative comparison, showing** Fourier power of the different images at a range of spatial frequencies, averaged across all 13 subjects. Images that are less smooth have more “salt and pepper” noise, and therefore have more power in the higher spatial frequencies. Error bars show the standard error of the mean, across the 13 subjects. The curves are statistically significantly different from each other (two-sample t-test, p<0.05) for spatial frequencies of 21 cycles per image and over.

The Fourier power spectra in Fig. 4b were calculated using the data of Lee et al. (2012) [15]. In each subject, fourteen axial slices from the middle of the brain encompassing Broca's area, were extracted. Each slice was padded with zeros to be 64×64 in size. The rotational average across orientations of the power in each slice was then calculated, using the Matlab function rotavg.m, written by Bruno Olshausen, and available online at http://redwood.berkeley.edu/bruno/ VS212B/ lab2/rotavg.m

## Results

### 1. GNB searchlight analyses produce smooth single-subject maps

Figure 4a shows illustrative slices from a single subject, in which it can be seen that the smoothest information maps arise from using GNB classifiers, which do not model covariance. SVMs, which do model covariance albeit with regularization, produce maps which are less smooth. Finally, LDA, which models covariance without any regularization, produces the least smooth maps of all. This result can be more formally quantified by calculating the Fourier power of the different images at a range of spatial frequencies (Figure 4b), averaging and calculating statistics across all 13 subjects and pooling across 14 axial slices as described in Methods section 5 above. Images which are less smooth have more “salt and pepper” noise, and therefore have more power in the higher spatial frequencies. The Fourier-power curves for the different classifiers are statistically significantly different from each other (two-sample t-test, p<0.05) for spatial frequencies of 21 cycles per image and over.

### 2. Replicated finding across two data sets: categorical processing in Broca's area

**Figure 5:**
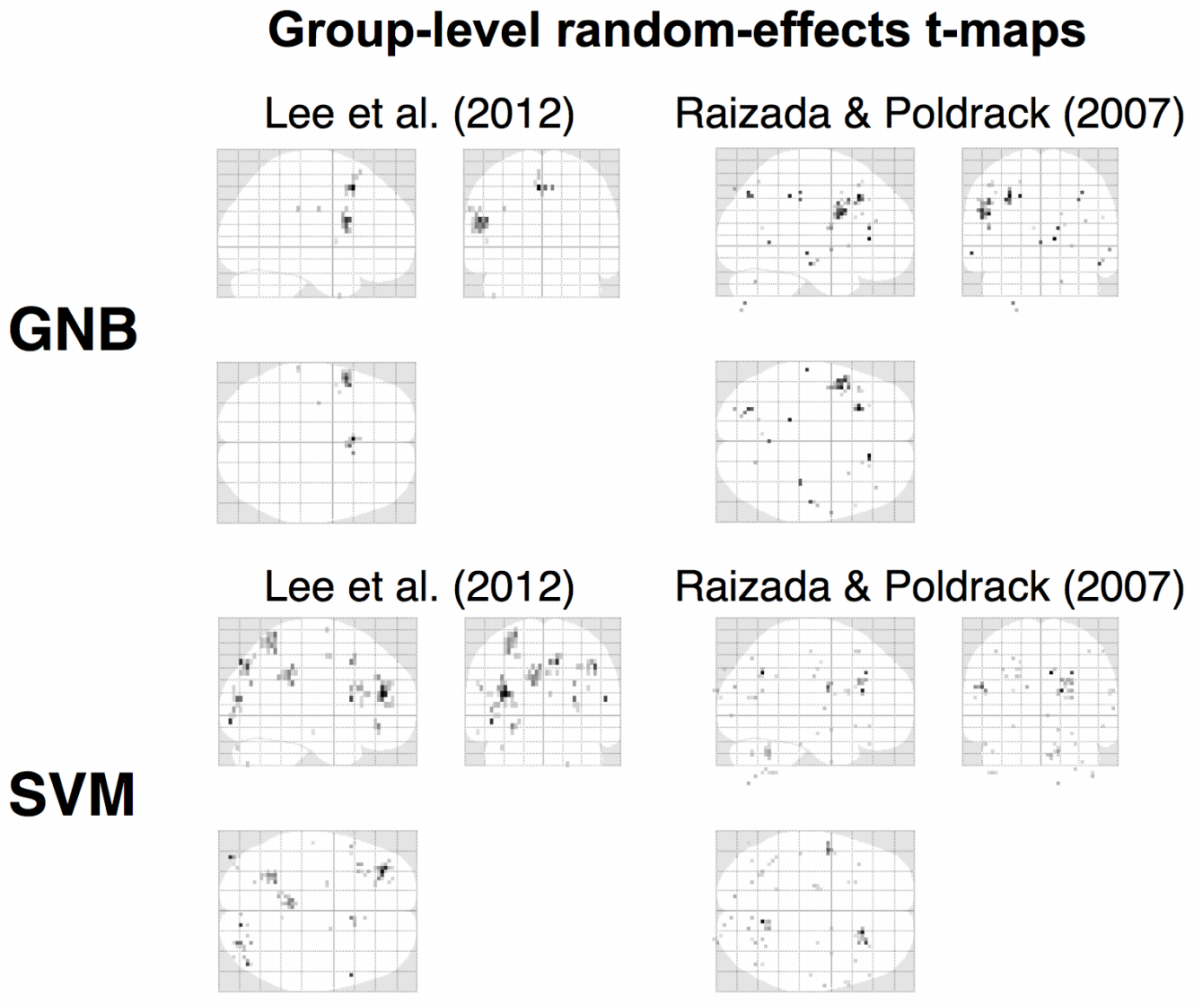
Comparison of GNB and SVM for group-level results. When searchlight maps use the GNB classifier, the group level analysis shows a clear ROI in Broca's area, in the two speech data sets. In this region, the patterns of fMRI activation contain information distinguishing between /ba/ and /da/ (upper two panels). In both data sets, Broca's ROIs are statistically robust, surviving multiple comparisons correction. By contrast, the SVM classifier does not produce results which replicate across the two data sets (lower panels). Group-level random effects maps are shown at p<0.001 uncorrected without any cluster-level thresholding (k = 0).

As Figures 5 shows, the two independent data sets yield remarkably convergent results: both show the brain carving up the phonetic continuum into the two perceptual categories of /ba/ and /da/ in the same region: Broca's area.

This Broca's cluster remained significant after correcting for multiple comparisons. For example, in Lee et al. (2012), the Broca's cluster has an FDR-corrected p-value of 0.001, and FWE-corrected value of 0.004. In Raizada & Poldrack (2007), the Broca's cluster has an FDR-corrected p-value of 0.006 and an FWE-corrected p-value of 0.013.

Finding Broca's area to be involved in speech perception is, from the standpoint of the present study, a reassuringly unsurprising result. Previous studies by other groups have found Broca's to be sensitive to phonetic categories, notably work by Myers and colleagues [18], [19]. Nonetheless, the similarities and differences between our MVPA study and the previous adaptation-fMRI study allow some inferences to be drawn about different spatial scales of phonetic processing across the brain: that topic is the focus of Lee et al., (2012).

For the purposes of the present study, we are interested in validating our proposed use of GNB classifiers in searchlight analyses which yields consistent results across two independent studies involving speech processing.

### 3. Do smoothed SVM searchlight-images end up producing the same group-level results as GNB images?

As the results above show, GNB searchlight analyses produced smooth single-subject information maps, and the group-level random effects analysis of these smooth maps yielded interpretable and replicable results across two different data sets.

By contrast, SVM searchlight analyses did not produce smooth single-subject maps, and led to group-level analyses which did not replicate across the two data sets.

This raises the question of whether the SVM analyses could yield GNB-like group-level results if the individual subjects' SVM-generated information maps had spatial smoothing applied to them before they are entered into group level random effects.

**Figure 6:**
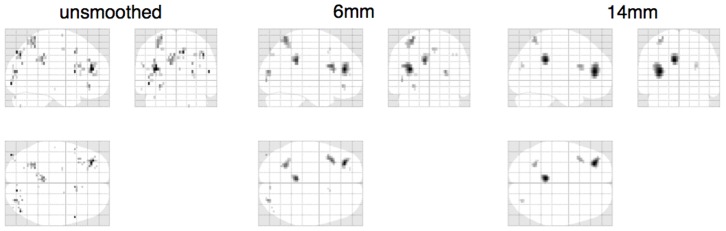
Group maps made from various size of smoothing kernels applied to data for SVM-searchlight. When smoothing is applied to the SVM-generated maps, the resulting random effects maps do not become similar to the GNB-generated map. Instead, the resulting maps simply look like slightly smoother versions of the random effects map generated from unsmoothed SVM images. This increased number of clusters makes the SVM analyses bear even less resemblance to the GNB analyses, regardless of the level of smoothing applied to them. Group-level random effects maps are shown at p<0.001 uncorrected without any cluster-level thresholding (k = 0).

Figure 6 shows that this is not the case. When smoothing is applied to the SVM-generated maps, the resulting random effects maps do not become similar to the GNB-generated map. Instead, the resulting maps simply, and perhaps unsurprisingly, look like slightly smoother versions of the random effects map generated from unsmoothed SVM images. In other words, the process of smoothing single-subject SVM images before entering them into the group-level analysis does not shift the locations of the resulting group-level clusters. These clusters stay centered in the same place, but simply end up becoming smoother.

Some recent studies using SVMs for searchlight analysis have applied spatial smoothing to the single-subject information maps, before entering them into the group-level random effects analysis [20]–[24]. The analyses here suggest that this processing step is unlikely to have produced the kind of group-level activations that are yielded by intrinsically smooth GNB-generated searchlight maps.

## Discussion

A classifier which declares itself to be naive in its very name is liable to have its worth underestimated. We have argued above that a GNB is often more powerful than one might expect, and that it is particularly well-suited for multi-subject searchlight fMRI studies, because it produces single-subject maps which are smooth. This smoothness helps different subjects' brains to be combined at the group level. We presented a heuristic theoretical argument for why a GNB would produce smooth maps, and then supported this with empirical findings [14], [15]. Collectively, these theoretical arguments and empirical results strongly suggest that the Gaussian Naive Bayes classifier is, despite its name, a non-naive choice for multi-subject searchlight fMRI studies.

### 1. A computational advantage of using GNBs: very fast analyses

An additional advantage of GNBs is that performing a searchlight analysis using a GNB is orders of magnitude faster than using other algorithms, such as SVMs or linear discriminants. As far as we are aware, this was first pointed out and computationally implemented by Pereira & Botvinick in Ref [25].

The reason for the GNB's speed is precisely because it does not model the covariance between voxels. After the log-probability values of the experimental conditions have been calculated for each individual voxel, they do not need to be recomputed when those voxels are combined together into different searchlight neighborhoods. The Naive Bayes log-probability of a given neighborhood is simply the sum of the log-probabilities of the voxels within that neighborhood.

By contrast, classifiers which do model the covariance between voxels must perform a new covariance computation for every different searchlight neighborhood. Thus, rather than simply summing together the results of prior computations, each searchlight requires the new calculation of an *n*-by-*n* covariance matrix, where *n* is the number of voxels in the searchlight neighborhood. For many classifier algorithms, such as linear discriminants and SVMs, this matrix must not only be calculated but also inverted. The larger the searchlight radius, the longer these computations require.

### 2. Relation to previous studies, and implications for future work

A number of previous studies have compared the performance of different types of classifiers in pattern-based fMRI analysis [25]–[31]. Two of those investigated using GNBs in searchlight analyses [25], [30], but neither of those two studies investigated the role of GNBs at the multi-subject level. As we have argued in this paper, it is only at the multi-subject level that the specific advantages of GNBs arise: the spatial smoothness of GNB-created single-subject maps becomes useful when combining slightly misaligned brains across subjects. As far as we are aware, the present paper is also the first in the neuroimaging literature to highlight results from machine learning showing that GNB can perform better than the “naive” in its name might suggest.

Of the studies that have used GNBs to analyze fMRI data, the classifiers have been used in a variety of different ways and in a variety of different contexts. Some of these studies have found GNB performance to be comparable to that of other classifiers [25], [27]. Other studies have shown GNBs to yield robust and neural plausible clusters of information-bearing activation [32], [33]. However, other studies have found GNB performance to be poor in comparison with other classifiers [29]–[31]. This diverse set of approaches and outcomes presents a confusing picture. In the present paper, we lay out a specific but common scenario in which GNBs would be expected to perform well: multi-subject searchlight studies. It is our hope that by providing theoretical arguments and empirical evidence in support of this claim, we may thereby help to explain and clarify the confusing diversity of findings relating to GNBs.

## References

[1] Demsar J (2006) Statistical comparisons of classifiers over multiple data sets. Journal of Machine Learning Research 7: 1–30.

[2] Thirion B, Pinel P, Meriaux S, Roche A, Dehaene S, et al (2007) Analysis of a large fMRI cohort: Statistical and methodological issues for group analyses. Neuroimage 35: 105–120. 17239619 10.1016/j.neuroimage.2006.11.054

[3] Mikl M, Marecek R, Hlustik P, Pavlicova M, Drastich A, et al (2008) Effects of spatial smoothing on fMRI group inferences. Magnetic Resonance Imaging 26: 490–503. 18060720 10.1016/j.mri.2007.08.006

[4] Li YM, Gilmore JH, Shen DG, Styner M, Lin WL, et al (2013) Multiscale adaptive generalized estimating equations for longitudinal neuroimaging data. Neuroimage 72: 91–105. 23357075 10.1016/j.neuroimage.2013.01.034 PMC3621129

[5] Kriegeskorte N, Goebel R, Bandettini P (2006) Information-based functional brain mapping. Proceedings of the National Academy of Sciences of the United States of America 103: 3863–3868. 16537458 10.1073/pnas.0600244103 PMC1383651

[6] Op de Beeck HP (2010) Against hyperacuity in brain reading: spatial smoothing does not hurt multivariate fMRI analyses? Neuroimage 49: 1943–1948. 19285144 10.1016/j.neuroimage.2009.02.047

[7] Bickel PJ, Levina E (2004) Some theory for Fisher's linear discriminant function, ‘naive Bayes’, and some alternatives when there are many more variables than observations. Bernoulli 10: 989–1010.

[8] Domingos P, Pazzani M (1997) On the optimality of the simple Bayesian classifier under zero-one loss. Machine Learning 29: 103–130.

[9] Hand DJ, Yu KM (2001) Idiot's Bayes - Not so stupid after all? International Statistical Review 69: 385–398.

[10] Ng Y, Jordan MI (2002) On discriminative vs. generative classifiers: A comparison of logistic regression and naive bayes. Advances in neural information processing systems 14: 841.

[11] Zhang H (2004) The optimality of naive Bayes. Proceedings of the 17th International FLAIRS conference 17.

[12] Raudys SJ, Jain AK (1991) Small sample size effects in statistical pattern recognition: Recommendations for practitioners. IEEE Transactions on pattern analysis and machine intelligence 13: 252–264.

[13] Hastie T, Tibshirani R, Friedman JH (2009) Elements of Statistical Learning: data mining, inference, and prediction (2nd ed.): Springer series in statistics. Springer. New York, NY: Springer.

[14] Raizada RDS, Poldrack RA (2007) Selective amplification of stimulus differences during categorical processing of speech. Neuron 56: 726–740. 18031688 10.1016/j.neuron.2007.11.001

[15] Lee YS, Turkeltaub P, Granger R, Raizada RDS (2012) Categorical Speech Processing in Broca's Area: An fMRI Study Using Multivariate Pattern-Based Analysis. Journal of Neuroscience 32: 3942–3948. 22423114 10.1523/JNEUROSCI.3814-11.2012 PMC6703443

[16] Friston KJ, Holmes AP, Worsley KJ, Poline JP, Frith CD, et al (1994) Statistical parametric maps in functional imaging: a general linear approach. Human brain mapping 2: 189–210.

[17] Mangasarian OL, Musicant DR (2001) Lagrangian support vector machines. The Journal of Machine Learning Research 1: 161–177.

[18] Myers EB (2007) Dissociable effects of phonetic competition and category typicality in a phonetic categorization task: An fMRI investigation. Neuropsychologia 45: 1463–1473. 17178420 10.1016/j.neuropsychologia.2006.11.005 PMC1876725

[19] Myers EB, Blumstein SE, Walsh E, Eliassen J (2009) Inferior Frontal Regions Underlie the Perception of Phonetic Category Invariance. Psychological Science 20: 895–903. 19515116 10.1111/j.1467-9280.2009.02380.x PMC2851201

[20] Bode S, Haynes JD (2009) Decoding sequential stages of task preparation in the human brain. Neuroimage 45: 606–613. 19111624 10.1016/j.neuroimage.2008.11.031

[21] Walther DB, Caddigan E, Fei-Fei L, Beck DM (2009) Natural Scene Categories Revealed in Distributed Patterns of Activity in the Human Brain. Journal of Neuroscience 29: 10573–10581. 19710310 10.1523/JNEUROSCI.0559-09.2009 PMC2774133

[22] Chen Y, Namburi P, Elliott LT, Heinzle J, Soon CS, et al (2011) Cortical surface-based searchlight decoding. Neuroimage 56: 582–592. 20656043 10.1016/j.neuroimage.2010.07.035

[23] Kahnt T, Heinzle J, Park SQ, Haynes JD (2011) Decoding different roles for vmPFC and dlPFC in multi-attribute decision making. Neuroimage 56: 709–715. 20510371 10.1016/j.neuroimage.2010.05.058

[24] Kahnt T, Heinzle J, Park SQ, Haynes JD (2010) The neural code of reward anticipation in human orbitofrontal cortex. Proceedings of the National Academy of Sciences of the United States of America 107: 6010–6015. 20231475 10.1073/pnas.0912838107 PMC2851854

[25] Pereira F, Botvinick M (2011) Information mapping with pattern classifiers: A comparative study. Neuroimage 56: 476–496. 20488249 10.1016/j.neuroimage.2010.05.026 PMC2975047

[26] Cox DD, Savoy RL (2003) Functional magnetic resonance imaging (fMRI) “brain reading”: detecting and classifying distributed patterns of fMRI activity in human visual cortex. Neuroimage 19: 261–270. 12814577 10.1016/s1053-8119(03)00049-1

[27] Mitchell TM, Hutchinson R, Niculescu RS, Pereira F, Wang XR, et al (2004) Learning to decode cognitive states from brain images. Machine Learning 57: 145–175.

[28] Zhang L, Samaras D, Tomasi D, Volkow N, Goldstein R (2005) Machine Learning for Clinical Diagnosis from Functional Magnetic Resonance Imaging. Proceedings of the 2005 IEEE Computer Society Conference on Computer Vision and Pattern Recognition (CVPR'05) - Volume 1 IEEE Computer Society. pp. 1211–1217.

[29] Ku SP, Gretton A, Macke J, Logothetis NK (2008) Comparison of pattern recognition methods in classifying high-resolution BOLD signals obtained at high magnetic field in monkeys. Magnetic Resonance Imaging 26: 1007–1014. 18691999 10.1016/j.mri.2008.02.016

[30] Misaki M, Kim Y, Bandettini PA, Kriegeskorte N (2010) Comparison of multivariate classifiers and response normalizations for pattern-information fMRI. Neuroimage 53: 103–118. 20580933 10.1016/j.neuroimage.2010.05.051 PMC2914143

[31] Schmah T, Yourganov G, Zemel RS, Hinton GE, Small SL, et al (2010) Comparing classification methods for longitudinal fMRI studies. Neural Computation 22: 2729–2762. 20804386 10.1162/NECO_a_00024

[32] Johnson JD, McDuff SG, Rugg MD, Norman KA (2009) Recollection, familiarity, and cortical reinstatement: a multivoxel pattern analysis. Neuron 63: 697–708. 19755111 10.1016/j.neuron.2009.08.011 PMC2771457

[33] Shinkareva SV, Malave VL, Mason RA, Mitchell TM, Just MA (2011) Commonality of neural representations of words and pictures. Neuroimage 54: 2418–2425. 20974270 10.1016/j.neuroimage.2010.10.042

